# Cerebellar and Prefrontal Structures Associated With Executive Functioning in Pediatric Patients With Congenital Heart Defects

**DOI:** 10.3389/fneur.2022.827780

**Published:** 2022-03-09

**Authors:** Daryaneh Badaly, Sue R. Beers, Rafael Ceschin, Vincent K. Lee, Shahida Sulaiman, Alexandria Zahner, Julia Wallace, Aurélia Berdaa-Sahel, Cheryl Burns, Cecilia W. Lo, Ashok Panigrahy

**Affiliations:** ^1^Learning and Development Center, Child Mind Institute, New York, NY, United States; ^2^Department of Psychiatry, University of Pittsburgh School of Medicine, Pittsburgh, PA, United States; ^3^Department of Radiology, UPMC Children's Hospital of Pittsburgh, Pittsburgh, PA, United States; ^4^Department of Biomedical Informatics, University of Pittsburgh School of Medicine, Pittsburgh, PA, United States; ^5^Department of Bioengineering, University of Pittsburgh School of Medicine, Pittsburgh, PA, United States; ^6^Traumatic Brain Injury Program, University of Pittsburgh Medical Center, Pittsburgh, PA, United States; ^7^Department of Developmental Biology, University of Pittsburgh, Pittsburgh, PA, United States

**Keywords:** cerebellum, prefrontal cortex, congenital heart defects (CHD), executive functioning, working memory, inhibition, mental flexibility

## Abstract

**Objective:**

Children, adolescents, and young adults with congenital heart defects (CHD) often display executive dysfunction. We consider the prefrontal and cerebellar brain structures as mechanisms for executive dysfunction among those with CHD.

**Methods:**

55 participants with CHD (*M* age = 13.93) and 95 healthy controls (*M* age = 13.13) completed magnetic resonance imaging (MRI) of the brain, from which we extracted volumetric data on prefrontal and cerebellar regions. Participants also completed neuropsychological tests of executive functioning; their parents completed ratings of their executive functions.

**Results:**

Compared to healthy controls, those with CHD had smaller cerebellums and lateral, medial, and orbital prefrontal regions, they performed more poorly on tests of working memory, inhibitory control, and mental flexibility, and their parents rated them as having poorer executive functions across several indices. Across both groups, there were significant correlations for cerebellar and/or prefrontal volumes with cognitive assessments of working memory, mental flexibility, and inhibitory control and with parent-completed ratings of task initiation, working memory, and planning/organization. Greater prefrontal volumes were associated with better working memory, among those with larger cerebellums (with group differences based on the measure and the prefrontal region). Greater prefrontal volumes were related to better emotional regulation only among participants with CHD with smaller cerebellar volumes, and with poorer inhibition and emotional regulation only among healthy controls with larger cerebellar volumes.

**Conclusion:**

The cerebellum not only contributes to executive functioning among young individuals with CHD but may also modulate the relationships between prefrontal regions and executive functioning differently for pediatric patients with CHD vs. health controls.

## Introduction

Advances in diagnostic, medical, and surgical techniques have dramatically improved the life expectancy of individuals with congenital heart defects (CHD), particularly among those with complex lesions requiring surgery early in life (1). With improved survival rates, greater attention has turned to the development and quality of life of those with CHD. Historically, clinicians and researchers have focused on anomalies in motor development, as these salient deficits appear early on (2). With medical advances resulting in successive cohorts surviving into adulthood, there has been a shift toward evaluating complex cognitive deficits, which are often not evident until school entry or later (3). Although the cognitive sequelae seen among children and adolescents with CHD are varied [for a review, see (4)], there appears to be a relative preservation of lower-level cognitive skills but risk for deficits in higher-order functions integrating and coordinating lower-level skills to achieve goals (5). With this context, executive functions—a set of abilities responsible for goal-directed activity—are of particular importance among pediatric patients with CHD. Importantly, executive functioning skills, which include working memory, inhibition of prepotent responses, and shifting between tasks and data streams among other skills (6), play a critical role in young individuals' psychological wellbeing, social adjustment, and scholastic and occupational success (7).

A number of studies have documented deficits in executive functioning skills among young individuals with CHD using standardized neuropsychological tests. In particular, children and adolescents with CHD, as compared to healthy controls, often have deficits in working memory, inhibitory control and mental flexibility among other skills [for a meta-analysis, see (8)]. Additionally, young persons with CHD are often described as displaying behavioral expressions of executive dysfunction using parental ratings [e.g., (9–12)]. Interestingly, work with an array of populations (13–16), including individuals with CHD (9–11), has consistently documented modest correlations between cognitive tests and behavioral ratings of executive functioning, suggesting that the different assessment methods offer both unique and overlapping information on development. In turn, prior studies have found that the different types of measures have both unique and overlapping associations with brain volume and cortical thickness (17, 18). Consequently, we examined both cognitive tests and behavioral ratings of executive functioning skills among children, adolescents, and young adults with CHD as compared to healthy peers.

Whereas the executive functioning deficits of children, adolescents, and young adults with CHD are increasingly well documented, the literature on the underlying mechanisms of such deficits is still emerging in many ways. Traditionally, deficits in executive functioning have been attributed to prefrontal dysfunction (19). In line with this suggestion, adolescents and adults with CHD have reduced prefrontal volumes (20, 21). Diminished white matter connectivity along the precentral sulcus has also been related to higher behavioral ratings of executive dysfunction and symptoms of attention-deficit/hyperactivity disorder (ADHD) among adolescents with CHD (22).

Empirical work has documented that higher order skills are not solely mediated by the prefrontal areas of the brain (23, 24). The cerebellum may also contribute to both early motor delays and later higher-order cognitive deficits among those with CHD. There is growing evidence that the cerebellum plays a key role in cognition and behavior (25), including executive functioning skills and behavioral symptoms of ADHD [for a review, see (26)]. The cerebellum has one of the highest regional brain blow flow requirements during the late gestational and early postnatal periods (27, 28) and, as such, may be susceptible to growth disturbances among those with CHD, who can be at risk for poor cerebral oxygen and substrate delivery early in life (29). Indeed, among infants, children, adolescents, and young adults with CHD, neuroimaging studies have found reductions in cerebellar volumes (21, 30, 31). In turn, cerebellar volumes are positively associated with working memory among adolescents with CHD (21) and inhibition, mental flexibility, and behavioral manifestations of executive functioning among young adults with CHD (30).

Interestingly, theoretical models have suggested that the cerebellum is key for the prefrontal system's development of higher-order thinking skills. For instance, Koziol et al. (32) argue that executive functions evolved from the need to anticipate and control behavior and that the cerebellum instructs the prefrontal systems on how to plan and problem solve by providing control mechanisms. Research similarly suggests that cerebellar development plays a critical role in the organization and development of downstream cortical structures, such as the prefrontal cortex. For example, Limperopoulos et al. (33) found that, among children who were born prematurely (with a mean age of 34 months), cortical growth was inversely related to the degree of early cerebellar injury. Moreover, research among neurodevelopmental and psychiatric populations has shown that structural cerebellar anomalies are associated with anomalous connectivity with frontal networks and differences in frontal volumes [e.g., (34, 35)]. Additionally, prefrontal and contralateral cerebellar regions activate in concert while performing executive function tasks (36). As such, cerebellar anomalies may modulate the role of prefrontal areas on executive functions, either as a function of early development or subsequent coordinated activity.

In the current study, we examined both the unique and interactive associations of prefrontal and cerebellar structures with executive functioning among children, adolescents, and young adults with CHD. We used multiple outcome measures, including paper-and-pencil and computerized tests of cognition and parental ratings of behavior. It was expected that both prefrontal and cerebellar structures would be associated with executive function, such that greater structural volumes would be associated with enhanced functioning. It was also anticipated that cerebellar volume would moderate associations between prefrontal volumes and executive functioning. However, we did not have *a priori* hypotheses regarding the direction of the moderation. If reduced cerebellar volumes are an indicator of abnormal early development across brain structures (as suggested by studies with children born preterm) (33), then the positive relationship between prefrontal areas and executive functioning may be more strongly evident with larger cerebellum. If reduced cerebellar volumes are compensated for by development of the prefrontal areas (as suggested by studies with children with autism spectrum disorder) (34), then the positive relationship between prefrontal areas and executive functioning may stronger with smaller cerebellum.

## Methods

### Participants

As part of a prospective study of brain development among children, adolescents, and young adults with CHD [previously described in (37)], we recruited 72 participants with varied heart lesions and 99 healthy peers between the ages of 6 and 25 years. Participants were recruited from a single center, using print and digital advertisements, an online registry of healthy volunteers, and referrals from targeted clinics. Participants underwent brain magnetic resonance imaging (MRI), completed neuropsychological testing, and provided demographic information and medical records. Participants who had reached the age of majority provided informed consent; minors were assented to the project, and their parent or legal guardian provided consent on their behalf. The project was approved by our Institutional Review Board (IRB) and completed in accordance with the ethical principles of the Helsinki Declaration.

Study exclusion criteria included comorbid genetic disorders, contraindications for MRI (e.g., a pacemaker), and non-English speakers. For healthy controls, study exclusion criteria also included preterm birth and neurological abnormalities (e.g., brain malformations, strokes, hydrocephalus). Participants with CHD were not excluded based on preterm birth and neurological abnormalities, as these factors are thought to occur more frequently as a function of CHD (38, 39). In addition to exclusion criteria, 17 participants with CHD and four healthy participants were not included in the final sample, as they did not complete brain imaging, the quality of their imaging was poor, or they did not complete any part of the neuropsychological testing. Our final sample therefore included 55 participants with CHD and 95 healthy peers. Of the final sample, 20 participants with CHD and 54 comparison peers had complete neuropsychological testing; data were not complete for all individuals, as participants elected not to complete parts of the evaluation or were ineligible for portions due to their age.

### Measures of Regional Brain Volumes

**Figure 1:**
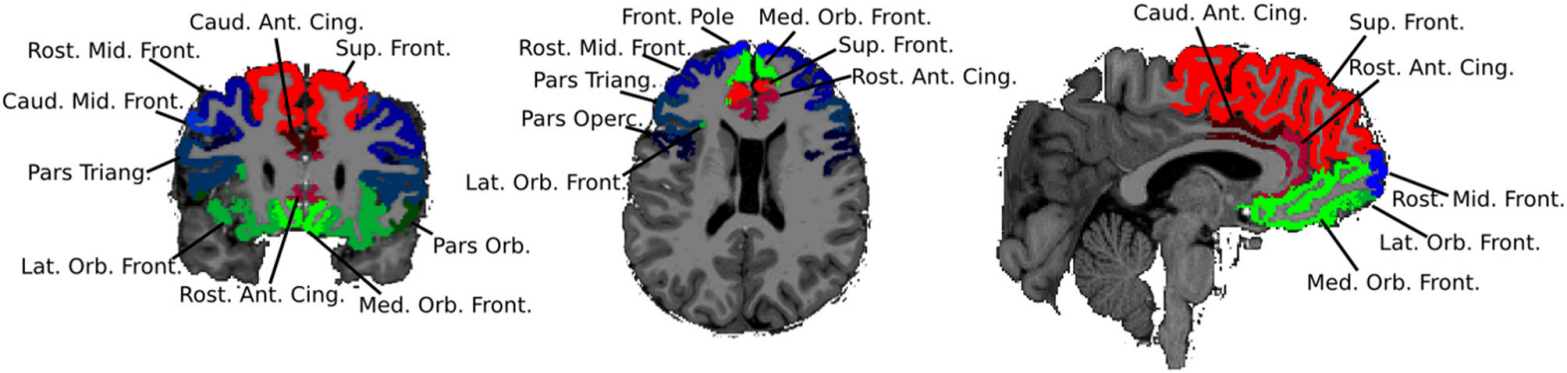
Subdivisions of the frontal lobe.

**Figure 2:**
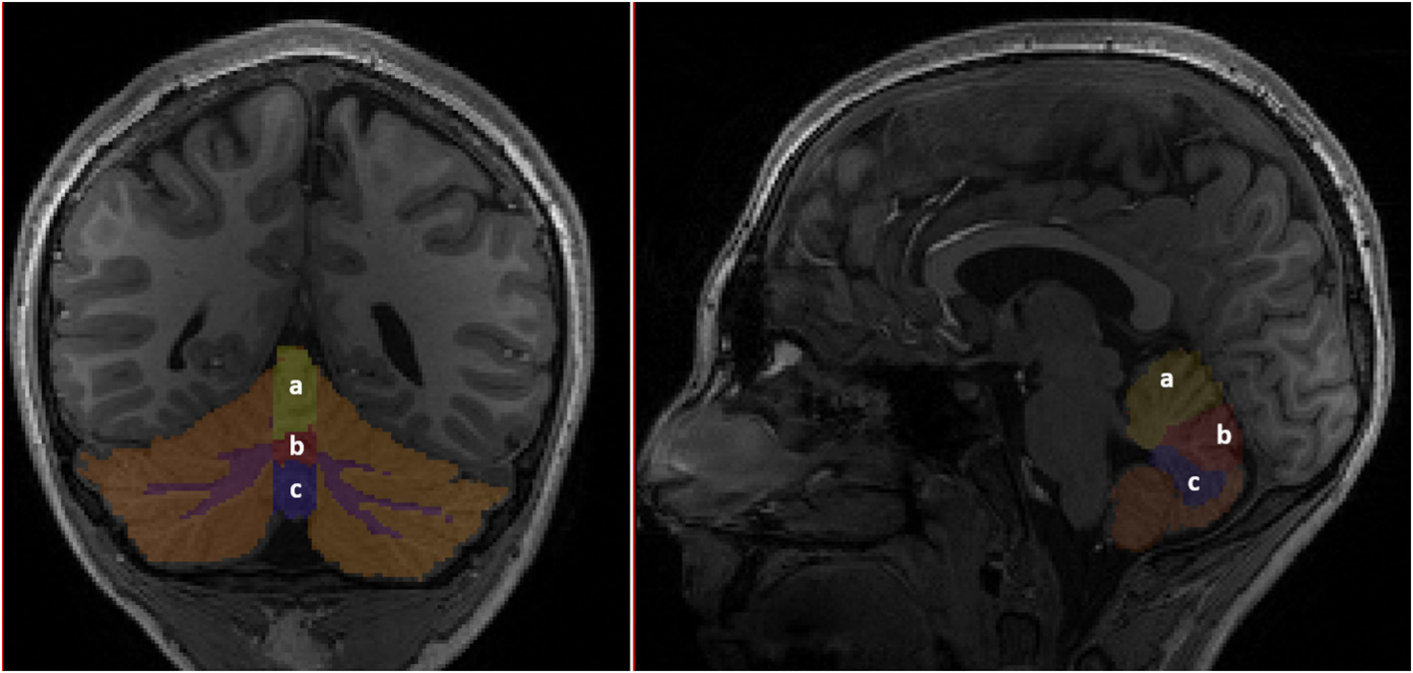
Subdivisions of the cerebellum. (a) Superior vermis. (b) Middle vermis. (c) Inferior vermis.

Participants underwent brain MRI on a 3 Tesla Skyra scanner (Siemens, Erlangen, Germany), using a 32-channel head coil. 3D sagittally acquired T1-weighted volumetric images were used as input for initial structural segmentation using the standard FreeSurfer pipeline (40). Automated cortical segmentation was done based on the Desikan Killiany Atlas, which subdivides the brain into 24 discrete cortical regions. For our analyses, we initially aggregated this parcellation into the specific cortical subdivisions of the prefrontal lobe (Figure 1) to examine structural differences between our two groups. In addition, the cerebellar vermis was manually segmented for each participant to obtain finer granularity than is provided by the FreeSurfer templates. The vermis was parcellated into superior, middle, and inferior segments (Figure 2). Although connections from the cerebellar hemispheres to the prefrontal cortex have been implicated in higher order cognition (41), we analyzed the vermis in detail, as (a) structural abnormities of the vermis have been documented among patients with CHD and associated with developmental outcomes (42, 43), and (b) vermal volumes show fewer changes across different age ranges both among typical developing youths and those with neurodevelopmental disorders characterized by executive functioning deficits (e.g., ADHD), which allows for comparisons in studies with a broad age range such as ours (44, 45). Following the segmentation, the total volume for each brain structure was extracted. Images and segmentations underwent secondary review by an experienced pediatric neuroradiologist, and any disagreements were resolved during a consensus review. No significant acquired or developmental brain abnormalities were noted in the cerebrum or cerebellum for our sample.

### Neuropsychological Measures

A trained technician supervised by an experienced neuropsychologist administered a battery of neuropsychological tests, which included both clinician- and computer-administered tests, providing multiple assessments of similar constructs. To assess general intellectual functioning, participants of all ages completed the Wechsler Abbreviated Scale of Intelligence, 2nd Edition (WASI-II) (46). We considered the four-subtest Full Scale IQ from the WASI-II, which provides a composite of verbal and non-verbal reasoning abilities.

To examine executive functioning, we first assessed working memory. Participants between 6 and 16 years old completed the Digit Span and Letter-Number Sequencing subtests of the Wechsler Intelligence Scale for Children Test, 4th Edition (WISC-IV) (47); the subtests assess abilities to attend to and manipulate in mind auditory information. Participants ages 7 and up also completed the List Sorting Working Memory Test from the NIH Toolbox (48); the subtest assesses working memory with auditory and visual stimuli. To further investigate executive functioning, participants ages 8 and up completed the Color-Word Interference Test (CWIT), the Trail Making Test (TMT), and the Verbal Fluency Test (VFT) from the Delis-Kaplan Executive Function System Test (D-KEFS) (49). We focused on trials of the subtests examining inhibition (CWIT Inhibition), sequencing skills and cognitive flexibility (TMT Number-Letter Switching), and cognitive flexibility and verbal fluency (VFT Category Switching). Participants of all ages were also administered the Flanker Inhibitory Control and Attention Test and the Dimensional Change Card Sort Test from the NIH Toolbox, measures of inhibitory control and cognitive flexibility, respectively.

### Parent-Completed Ratings

Parents completed behavioral ratings of executive functioning on the Behavior Rating Inventory of Executive Function Test (BRIEF) (50), which is available for children and adolescents ages 5–18. Validity indices on the BRIEF were acceptable for all participants. We examined the eight subscales of the BRIEF. Higher scores on the BRIEF denote greater parent-reported concerns for deficits in executive functioning.

### Analysis Plan

We first examined group differences between pediatric patients with CHD and healthy controls. To analyze group differences in patient demographics, we conducted independent sample *t*-tests for continuous variables (such as age) and χ^2^-tests for frequencies within categorical variables (such as racial background). We compared the regional prefrontal and cerebellar volumes between the two groups using analysis of covariance (ANCOVA) with a control for age, adjusting for false discovery rate using Benjamini and Hochberg's (51) method. We examined group differences on measures of executive functions using independent sample *t*-tests, with a control for false discovery rate. Because scores on tests of executive functioning are standardized by age, we used *t*-tests rather than an ANCOVA controlling for age.

To examine the associations between cerebellar volumes, prefrontal volumes, and executive functioning, we used bivariate correlations and multiple regression analyses. Such linear analyses assume a number of data attributes (52). The methods assume that the relationship between the predictor or predictors and the outcome is linear, that the residuals are distributed with equal variance across the range of the outcome variable, and that the residuals are normally distributed. To check these assumptions, for each of our models, we inspected plots of the residuals against the predicted values, and we examined univariate statistics for the residuals and inspected their normal probability plots and histograms. For models with skew, we applied transformations to normalize the distributions. As the transformations did not alter the overall pattern of results, for ease of discussion, analyses with untransformed scores are presented below. In addition, because collinearity can cause numerical problems that produce invalid regression estimates (52), we examined the relations among our predictor variables. Substantial collinearity, indicated by tolerances <0.10 and variance inflation factors >10.00, was not found for any of our models.

We examined the bivariate correlations of the cerebellar volume and the prefrontal volumes with executive functioning. To explore group differences in the correlations between patients with CHD and healthy controls, we used Fisher (53, 54)'s *r*-to-*z* transformation.

Next, we specified multiple linear regression models for each executive functioning outcome examining the interactive effects of cerebellar and prefrontal volumes, as detailed in the equations below. Separate models were specified for each pairing of subregions of the brain. Specifically, main effects for group (i.e., patient with CHD vs. healthy control), cerebellar volume, and prefrontal volume were entered on step 1. The interaction for cerebellar volume by prefrontal region was entered on step 2. The three-way interaction for group by cerebellar volume by prefrontal region was then entered on step 3. To decompose significant interactions with continuous moderators, we followed the recommendations of Aiken and West (55), testing simple slopes after algebraically fixing the variable to high, median, and low levels (i.e., 1 standard deviation above the mean, the mean, and 1 standard deviation below the mean).

*Step 1: Y* (Executive Function) = *B_0_ + B_1_X_1_* (Group) + *B_2_X_2_* (Cerebellar Volume) + *B_3_X_3_* (Prefrontal Volume) + ε

*Step 2: Y* (Executive Function) = *B_0_ + B_1_X_1_* (Group) + *B_2_X_2_* (Cerebellar Volume) + *B_3_X_3_* (Prefrontal Volume) + *B_4_X_2_ X_3_* (Cerebellar Volume × Prefrontal Volume) + ε

*Step 3: Y* (Executive Function) = *B_0_ + B_1_X_1_* (Group) + *B_2_X_2_* (Cerebellar Volume) + *B_3_X_3_* (Prefrontal Volume) + *B_4_ X_1_X_2_ X_3_* (Group × Cerebellar Volume × Prefrontal Volume) + ε

## Results

### Group Differences

Participant demographics are detailed in Table 1. Although participants with CHD had a slightly lower level of intellectual functioning compared to healthy controls, they generally performed within normal limits on tests of intelligence, similar to prior research (9–11). Of note, even though the intellectual level of healthy controls in the current study differed from the population mean (*t* = 7.98, *p* < 0.001), so did youths with CHD (*t* = 2.03, *p* < 0.05), suggesting that we captured typical group differences even if participants were slightly higher functioning than would be expected. In addition, there were larger percentages of individuals who identified as White and whose parents completed at least a bachelor's degree among participants with CHD as compared to healthy controls. Otherwise, the two groups of participants did not differ on demographic variables.

**Table 1:** Demographic differences between youth with CHD and healthy controls.

	**Healthy controls**	**Youths with CHD**	***p***
Mean age (SD)	13.13 (3.75)	13.93 (4.40)	0.239
Percent male	50.5	63.6	0.120
Percent right handed	74.7	78.2	0.208
Percent white	62.1	87.3	0.001
Percent college-educated parent	48.4	14.5	<0.001
Mean WASI-II FSIQ (SD)	110.65 (12.98)	104.19 (15.34)	0.046

*WASI-II FSIQ, Wechsler Abbreviated Scale for Intelligence, 2nd Edition Full Scale Intelligent Quotient*.

We compared the regional prefrontal and cerebellar volumes between pediatric patients with CHD and healthy peers using ANCOVA controlling for age (Table 2). Participants with CHD had significantly smaller overall cerebellar volume than healthy controls. However, there were no significant group differences for the vermis or the regions of the vermis, contrasting prior research with fetal and infant populations (42, 43). Prefrontal regions, with the exception of the pars triangularis and the lateral orbitofrontal region, were also significantly smaller among participants with CHD as compared to healthy controls.

**Table 2:** Cerebellar and prefrontal region structural differences between youth with CHD and healthy controls.

**Structure**	**Healthy controls**		**Youths with CHD**		**F**
	**Mean**	**SD**	**Mean**	**SD**	
**Cerebellar regions**					
Superior vermis	6,787	1,305	6,493	1,251	2.52
Middle vermis	4,180	865	3,919	667	4.01
Inferior vermis	3,643	776	3,775	675	0.88
Total vermis	14,610	2,631	14,187	2,281	1.36
Total cerebellum	108,394	12,552	101,891	11,830	8.83^*^
**Lateral prefrontal regions**					
Pars opercularis	8,162	1,192	7,773	1,223	5.32^*^
Pars triangularis	6,656	1,240	6,794	1,405	0.49
Rostral middle frontal	25,229	4,219	23,248	5,031	9.80^*^
Caudal middle frontal	7,117	1,338	6,634	1,380	5.09^*^
Total lateral prefrontal	47,163	6,442	44,447	7,597	7.94^*^
**Medial prefrontal regions**					
Caudal anteriorcingulate	6,634	1,380	6,379	1,097	9.17^*^
Rostral anteriorcingulate	5,906	871	5,853	863	6.11^*^
Superior frontal	39,226	4,990	37,167	5,347	7.27^*^
Total medial prefrontal	51,458	6,316	48,602	6,444	9.08^*^
**Orbital prefrontal regions**					
Pars orbitalis	2,277	432	2,134	570	4.83^*^
Lateral orbitofrontal	1,571	301	1,491	360	2.71
Medial orbitofrontal	2,660	534	2,504	461	4.31^*^
Total orbital prefrontal	6,508	809	6,130	1,007	8.81^*^

* *F-tests which were significant at the 0.05 level with a control for false discovery rate conducted separately for cerebellar regions and frontal regions*.

*Models controlled for age. Volumes are in mm^3^*.

We also examined group differences on tests of executive functioning (Table 3). Patients with CHD performed more poorly on a test of working memory from the WISC-IV (Letter-Number Sequencing), a test of inhibitory control from the D-KEFS (CWIT Inhibition), and tests of mental flexibility on the D-KEFS (TMT Number-Letter Switching) and the NIH Toolbox (Dimensional Change Card Sorting). Parental ratings of executive functioning (with the exceptions of inhibition and organization of materials) differed between participants with CHD and comparison peers, such that those with CHD displayed more executive dysfunction.

**Table 3:** Executive functioning differences between youth with CHD and healthy controls.

	**Healthy controls**		**Youths with CHD**		
**Test**	**Mean**	**SD**	**Mean**	**SD**	***t***
**WISC-IV**					
Digit Span	10.42	2.76	9.32	2.75	1.93
Letter-Number Sequencing	10.95	2.01	9.51	2.43	3.21^*^
**D-KEFS**					
TMT Number-Letter Switching	11.15	1.97	8.61	2.69	5.34^*^
VFT Category Switching, Correct Responses	11.22	3.53	10.00	2.54	1.79
VFT Category Switching, Switching Accuracy	11.25	3.43	10.73	2.38	0.79
CWIT Inhibition	10.67	2.42	8.83	3.05	2.88^*^
**NIH Toolbox**					
List Sorting Working Memory	105.70	13.11	102.55	16.04	1.27
Flanker Inhibitory Control and Attention	105.70	14.29	95.79	14.54	1.94
Dimensional Change Card Sorting	102.55	16.40	96.23	16.63	2.21^*^
**BRIEF**					
Inhibit	46.54	7.53	49.49	9.63	−1.79
Shift	44.97	8.03	51.57	13.47	−3.29^*^
Emotional Control	44.32	10.34	49.49	11.67	−2.41^*^
Initiate	47.67	10.25	53.89	12.64	−2.82^*^
Working Memory	48.70	9.77	56.70	13.30	−3.65^*^
Plan/Organize	47.32	9.87	55.51	12.80	−3.80^*^
Organization of Materials	48.53	9.82	52.73	12.31	−1.98
Monitor	44.80	9.34	51.46	12.36	−3.22^*^

* *t-Tests which were significant at the 0.05 level with a control for false discovery rate*.

*Scores are expressed as scaled scores on the WISC-IV and D-KEFS (mean of 10 and standard deviation of 3), as standard scores on the NIH Toolbox (mean of 100 and standard deviation of 15), and as T-scores on the BRIEF (mean of 50 and standard deviation of 10)*.

### Bivariate Correlations

Next, we examined the bivariate correlations among prefrontal and cerebellar regions. Across the groups of participants, there was structural covariance between total cerebellar volume and each of the prefrontal regions (*r*s = 0.29–0.53; *p* < 0.05), with the exception of the caudal middle frontal regions among participants with CHD (*r* = 0.12; *p* = 0.39). Among participants with CHD, there was also structural covariance between the middle vermis and the superior frontal region (*r* = 0.28; *p* < 0.05). Among healthy controls, there was structural covariance between each of the cerebellar regions and both the pars opercularis (*r*s = 0.23–0.43; *p* < 0.05) and the pars orbitalis (*r*s = 0.22–0.35; *p* < 0.05). Given our interest in the prefrontal regions in relation to cerebellar maturation, we focused our subsequent analyses on the total volumes for the cerebellum, lateral prefrontal region, medial prefrontal region, and orbital prefrontal region rather than subdivisions of the brain regions, based on the initial findings that the overarching regions best captured covariance among the brain structures.

We then examined the correlations of the cerebellar volume and the prefrontal volumes (i.e., lateral prefrontal region, medial prefrontal region, and orbital prefrontal region) with executive functioning (Table 4). Based on Fisher's *r*-to-*z* transformations, correlations did not statistically significantly differ between participants with CHD and healthy controls; as such, we discuss the pattern of associations across groups. The cerebellum, lateral prefrontal region, and medial prefrontal region were positively associated with working memory on the WISC-IV Letter-Number Sequencing; similarly, the cerebellum and the medial prefrontal region were positively associated with working memory on the NIH Toolbox List Sorting Working Memory. There were positive correlations between the cerebellar, lateral prefrontal, and orbital prefrontal volumes and mental flexibility on the D-KEFS Trail Making Test, and there were positive correlations between each of the prefrontal volumes and mental flexibility on the NIH Toolbox Dimensional Change Card Sorting. Greater cerebellar and prefrontal volumes were associated with greater inhibitory control on both the D-KEFS Color-Word Interference Test and the NIH Toolbox Flanker Inhibitory Control and Attention. On ratings from participants' parents, greater cerebellar, lateral prefrontal, and medial prefrontal volumes were related to better working memory (BRIEF Working Memory). In addition, greater cerebellar volume was related to better task initiation and planning/organization (BRIEF Working Memory, Plan/Organize).

**Table 4:** Correlations of cerebellar and prefrontal regions with executive functioning.

	**Brain region**			
**Test**	**Cerebellum**	**Lateral prefrontal**	**Medial prefrontal**	**Orbital prefrontal**
**WISC-IV**				
Digit Span	0.132	0.192	0.183	0.193
Letter-Number Sequencing	0.243^*^	0.271^**^	0.256^*^	0.177
**D-KEFS**				
TMT Number-Letter Switching	0.281^**^	0.213^*^	0.150	0.258^*^
VFT Category Switching, Correct Responses	0.035	0.017	0.064	0.104
VFT Category Switching, Switching Accuracy	0.017	0.001	0.035	0.081
CWIT Inhibition	0.337^**^	0.255^*^	0.268^*^	0.258^*^
**NIH Toolbox**				
List Sorting Working Memory	0.241^**^	0.097	0.224^**^	0.120
Flanker Inhibitory Control and Attention	0.238^**^	0.232^**^	0.245^**^	0.215^*^
Dimensional Change Card Sorting	0.128	0.230^**^	0.198^*^	0.226^**^
**BRIEF**				
Inhibit	−0.171	−0.081	−0.067	0.039
Shift	−0.192	−0.063	−0.051	0.002
Emotional Control	−0.112	−0.057	−0.018	−0.042
Initiate	−0.237^*^	−0.135	−0.084	−0.160
Working Memory	−0.254^**^	−0.232^*^	−0.198^*^	−0.161
Plan/Organize	−0.241^*^	−0.176	−0.152	−0.138
Organization of Materials	−0.129	−0.022	0.000	−0.033
Monitor	−0.153	−0.168	−0.112	−0.064

* *p < 0.05*.

** *p < 0.01*.

### Hierarchical Regressions

To examine the moderating role of the cerebellum on associations between prefrontal regions and executive functioning, we specified linear regression models for each executive functioning outcome examining the interactive effects of cerebellar and prefrontal volumes. Acknowledging that interpretive caution is needed, we discuss interactive regression effects significant at the 0.10 level. Because detecting significant interaction terms requires vastly greater sample sizes than main effects, considering a less restrictive threshold of significance can help identify meaningful effects in smaller samples. With this caveat in mind, significant interactions emerged for both lateral prefrontal and orbital prefrontal volumes with cerebellar volume for working memory on the WISC-IV Digit Span and for lateral prefrontal volume with cerebellar volume for working memory on the WISC-IV Letter-Number Sequencing. Greater lateral prefrontal volume was associated with better working memory, among participants with larger cerebellar volumes (WISC-IV Digit Span: ß = 0.360; *p* = 0.022; WISC-IV Letter-Number Sequencing: ß = 0.339; *p* = 0.023) but not smaller ones (WISC-IV Digit Span: ß = −0.156; *p* = 0.345; WISC-IV Letter-Number Sequencing: ß = −0.052; *p* = 0.741). Although there was a similar pattern of findings for orbital prefrontal volume and working memory on the WISC-IV Digit Span at high levels (ß = 0.241; *p* = 0.087) and low levels (ß = −0.167; *p* = 0.294) of cerebellar volume, the interaction differed by group. Greater orbital prefrontal volume was related to better working memory, only among healthy controls with larger cerebellum (ß = 0.446; *p* = 0.029).

There were several significant three-way interactions for group by cerebellar volume by prefrontal region for behavioral ratings of executive functioning, as depicted in Table 5. However, the decomposition of simple slopes only revealed significant results for a subset of interactions, on which we will focus on our discussion. There was a negative relationship between medial prefrontal volume and difficulties with working memory, which decreased in strength from high (ß = −0.514; *p* = 0.142) to medium (ß = −0.383; *p* = 0.046) to low levels of cerebellar volume (ß = −0.251; *p* = 0.328), only among participants with CHD. Only among healthy controls with larger cerebellar volumes, greater orbital prefrontal volume was associated with greater difficulties with inhibition (ß = 0.403; *p* = 0.031), and greater medial prefrontal volume was associated with greater difficulties with emotional regulation (ß = 0.394; *p* = 0.041). There was a negative association between lateral prefrontal volume and difficulties with emotional regulation, which was only significant among participants with CHD with smaller cerebellar volume (ß = −0.693; *p* = 0.025).

**Table 5:** Interactive associations of cerebellar volume and prefrontal volumes with executive functioning.

**Test**	**Step**	**Prefrontal region in model**	**ß**	***p***
**WISC-IV**				
Digit Span	2	Lateral prefrontal	−3.274	0.028
	2	Orbital prefrontal	−2.767	0.035
	3	Orbital prefrontal	−1.263	0.057
Letter-Number Sequencing	2	Lateral prefrontal	−2.478	0.079
**BRIEF**				
Inhibit	3	Lateral prefrontal	−1.028	0.063
	3	Medial prefrontal	−1.276	0.028
	3	Orbital prefrontal	−1.544	0.014
Emotional Control	3	Lateral prefrontal	−1.151	0.035
	3	Medial prefrontal	−0.970	0.093
Initiate	3	Lateral prefrontal	−0.894	0.096
Working Memory	3	Lateral prefrontal	−0.906	0.078
	3	Medial prefrontal	−0.940	0.085
	3	Orbital prefrontal	−1.077	0.074
Monitor	3	Lateral prefrontal	−1.040	0.053
	3	Medial prefrontal	−1.100	0.055

*Only effects significant at the p < 0.10 level are shown. There were no significant effects at the p < 0.10 level on step 2 or 3 for the models with the following outcomes: D-KEFS TMT-Number-Letter Sequencing, VFT-Category Switching, Correct Responses, VFT-Category Switching, Switching Accuracy, and CWIT-Inhibition; NIH Toolbox List Sorting Working Memory, Flanker Inhibitory Control and Attention, and Dimensional Change Card Sorting; BRIEF Shift, Plan/Organize, and Organization of Materials*.

Lastly, to account for variation in brain volume across the age range, we reran our linear models partialling out age. As there was a similar pattern of findings, we present the unadjusted models. Given the significant difference in intelligence among participants with CHD and healthy controls, we also reran our models with intellectual functioning as a control. Again, similar findings emerged. Because tests of intelligence incorporate aspects of executive functioning (i.e., reasoning and problem solving) and are highly related to measures of executive functions (6), we presented the models without the control for intellectual functioning (which would statistically remove an aspect of executive functioning from analyses). Indeed, it has been previously argued that controlling for intellectual quotient when examining neuropsychological skills (such as executive functioning) among those with neurodevelopmental risk factors (such as those with CHD) is methodologically tenuous because decrements in overall ability are expected, rendering statistical control impossible (56).

## Discussion

Children and adolescents with CHD, particularly those with cyanosis or those requiring surgical intervention in the first year of life, often present with deficits in executive functioning. Traditionally, such deficits have been attributed to prefrontal dysfunction. Increasingly, research has shown that higher order cognitive skills and their behavioral manifestations are not solely mediated by the prefrontal areas of the brain, and the cerebellum may also play an important role. Thus, we examined the unique and interactive associations of cerebellar and prefrontal structures on executive functioning among patients with CHD as compared to healthy peers. Notably, we focused on school-age children, adolescents, and young adults, rather than younger children. Because of the dynamic nature of development, assessments conducted with younger children (which may be limited in scope) often have limited predictive validity for later adjustment (57). The impact of certain deficits may only be apparent among older children, such as late-maturing executive functioning skills (58).

We first examined the structural differences in the cerebellar and prefrontal regions among patients with CHD and healthy controls. Compared to healthy controls, participants with CHD had smaller cerebellums as well as smaller lateral prefrontal, medial prefrontal, and orbital prefrontal regions. Prior research has similarly found that prefrontal surface area and cerebellar volume is reduced among adolescents and young adults with CHD compared to healthy peers (21, 30). As such, our findings contribute to the growing literature that cardiac anomalies are associated with dysmaturation of brain regions responsible for higher order cognitive functions, likely as a result of reductions in cerebral oxygenation and shared genetic factors (59). We also examined differences in executive functioning among participants with CHD and healthy controls. Overall, both groups generally performed within normal limits on cognitive measures and behavioral indices of executive functioning, likely reflecting the higher functioning nature of our sample. However, those with CHD had poorer executive functioning on certain cognitive measures and worse executive functioning on certain behavioral indices than their healthy peers, similar to prior research (9–11). Importantly, such differences in executive functioning place pediatric patients with CHD at greater risk for socioemotional and adaptive maladjustment throughout their development (7).

We examined the associations of cerebellar and prefrontal structures on executive functioning outcomes among patients with CHD and healthy controls. The cerebellum is thought to play a role in cognitive and behavioral regulation starting early in life and persisting into childhood and adolescence. Indeed, prior research has found that, among newborns with acyanotic heart lesions, reduced cerebellar volume is related to poorer behavioral state regulation (60), and, among adolescents and young adults with CHD, cerebellar volumes are associated with working memory, inhibition, mental flexibility, and behavioral manifestations of executive functioning (21, 30). Similarly, the current study, which included children, adolescents, and young adults with CHD, found that cerebellar volume was associated with cognitive assessments of working memory, inhibitory control, and mental flexibility as well as behavioral ratings of executive functioning (i.e., task initiation, working memory, and planning/organization). In line with the extant literature [e.g., (6, 19)], there were also associations for prefrontal areas with different measures of executive functioning. Interestingly, though, studies with patients with CHD have not consistently revealed associations between prefrontal volumes and executive functions [e.g., (21)], underscoring the need for a more refined considerations of brain correlates.

With this in mind, we examined the interactive associations of cerebellar and prefrontal structures on executive functioning. Fronto-cerebellar connectivity has long been documented, with the lateral part of the prefrontal cortex connecting to the cerebellum *via* pontine nuclei and the cerebellum sending projections back to the prefrontal cortex *via* the dentate nucleus and thalamus (61). Given this cortico-cerebellar loop, the cerebellum may play an important role in modulating the relationship between prefrontal regions and executive functioning (62, 63). Indeed, across patients with CHD and their healthy peers, our findings suggested that prefrontal volumes are positively associated with working memory, particularly among those with larger cerebellar volumes. In line with our findings, it has been suggested that the cerebellum may be recruited in supporting the prefrontal cortex when tasks require more cognitive resources (e.g., greater working memory) (63). Among young individuals, larger cerebellar volumes may translate to greater support for prefrontal networks, which can in turn more efficiently tackle working memory demands.

Prior empirical and theoretical work has suggested that the cerebellum may be related to not only executive functions but also socioemotional processes. Schmahmann proposed the cerebellar cognitive affective syndrome (CCAS) (64), which describes the cerebellum's involvement in not only cognitive processes but also affective and social regulation. Meta-analyses have documented a key role of the cerebellum in emotional processing (65) and social cognition (66), and those with CHD show higher rates of emotional distress (67) and impairments in social cognition (68). Research furthermore suggests that dysmaturation and early injury affecting the cerebellum may affect the maturation of neocortical regions and their functional impact on socioemotional processes (69). In line with this framework, we found that cerebellar volume was associated with emotional regulation, it moderated the relations between prefrontal volumes and emotional regulation, and moderation effects differed across participants with CHD and healthy controls (which likely differed in early cerebellar structure). That being said, future research is needed to better understand the role of the cerebellum not only on emotional functioning but also on social cognition, behavior, and adjustment among patients with CHD.

We described one of the first studies using a computerized screener of cognitive functioning (namely, the NIH Toolbox) among young individuals with CHD. Prior research has found that measures of cognitive skills, including executive functioning, from the NIH Toolbox have appropriate convergent and divergent validity with traditional paper-and-pencil measures (70). Given such sound psychometric properties, there has been growing interest in developing and using computerized tools (e.g., CANTAB, CogState, Impact, NIH Toolbox) to screen for cognitive dysfunction and track changes over time among medically complex individuals in clinical and empirical settings (71). While computerized assessments do not provide as detailed information as comprehensive neuropsychological evaluations, they may help identify those who would benefit from such assessment clinically, and they can provide a more temporally and fiscally efficient avenue for researchers. The results of the present investigation, along with emerging work from other research groups (72, 73), suggest that the subtle cognitive deficits seen among patients with CHD may, in part, be detected by a computerized assessment.

### Limitations and Future Directions

The main limitation of our study is its sample size. Although our sample was larger than those found in prior studies examining the role of cerebellar volume on executive functioning among patients with CHD (21, 30), we had limited power to detect effects within complex models. As a result, we elected to use a less stringent index of significance when interpreting the findings of our regression models with interactive effects. We also limited the scope of our analyses. For example, there was limited power to examine the moderating effect of age, although there is a maturation of the prefrontal cortex and cerebellum and a refinement of executive functions throughout childhood and into young adulthood (74, 75). We furthermore did not explore the impact of biological and environmental factors on associations between the cerebellum and cognitive and behavioral functioning. For instance, participants' type of heart lesion (e.g., single vs. double ventricle; cyanotic vs. acyanotic), their peri-operative complications, and their socioeconomic status might affect both their brain development and functional outcomes. Indeed, differential associations between cerebellar structure and behavioral regulation have been found among newborns with different heart lesions (60). It will be important to not only replicate our findings within larger samples (confirming that they are not the spurious result of a small sample) but also extend analyses to additional brain regions, confounds, and moderators.

A second limitation of our study is its cross-sectional design. Although a cross-sectional design allows one to draw conclusions about the relations between different brain structures and functional outcomes, it cannot speak to changes in functioning or brain development. Studies with longitudinal designs can characterize the changes in the development of distinct executive skills over time and the cortical-cerebellar system's role in this progression. It should be noted, though, that few programs have been able to examine longitudinal associations among neuroradiological and neuropsychological findings, given the more recent recognition of the importance of brain development and quality of life among those with CHD. The Boston Circulatory Arrest Study and a handful of others offer notable exceptions (22).

Although our results suggest that the cerebellum may affect the relationship between prefrontal regions and executive functioning among patients with CHD, our project focused on the structure of the brain rather than connections among different regions or their coordinated activation (areas of study that may provide further insight into the mechanisms for executive dysfunction among those with CHD). As such, future research should explore how indices of anatomical connectivity (e.g., measured with diffusion tensor imaging) and functional connectivity (e.g., measured with resting state temporal correlations) between the cerebellum and prefrontal regions are associated with executive functioning outcomes (76). Such investigations may be particularly important, as it has been argued that the functional impact of injury and atypical maturation of the cerebellum (as seen among those with CHD) is more so due to its connections to extracerebellar regions than its intrinsic characteristics (33, 77). It may also be important to explore the activation of the brain regions during tasks of executive functioning (e.g., with functional magnetic resonance imaging) (78). Indeed, prior research suggests that cerebellar activation during working memory tasks consistently increases with load and may decrease during challenging tasks from childhood to adulthood, suggesting the cerebellum supports the prefrontal cortex when cognitive demands are high and when fronto-parietal networks are specializing (63). Cerebellar dysmaturation among those with CHD may thus disrupt these normal processes, subtly affecting working memory abilities.

## Conclusion

In conclusion, we examined the associations of cerebellar and prefrontal structures on executive functioning among patients with CHD as compared to healthy controls. The study included multiple neuropsychological outcome measures, including paper-and-pencil and computerized tests of cognition and parental ratings of associated behaviors. It has previously been highlighted that the cerebellum shares bidirectional connections with the prefrontal cortex, has been implicated executive processes, and is thought to have a refining or modulating role on cognitive functions (63). Current results not only echoed that the cerebellum contributes to executive functioning among young individuals with CHD but also provided the first evidence among those with CHD that the cerebellum may modulate the relationship between prefrontal regions and cognitive and behavioral indices of executive functioning.

## Data Availability Statement

The raw data supporting the conclusions of this article will be made available by the authors, without undue reservation.

## Ethics Statement

The project was reviewed and approved by the Institutional Review Board (IRB) at the University of Pittsburgh. Written informed consent to participate in this study was provided by participants or their parent/legal guardian.

## Author Contributions

DB analyzed and interpreted the data and drafted and revised the manuscript. SB and RC managed different aspects of the data collection, contributed to the initial conceptualization of the manuscript, and provided comments on the manuscript. VL analyzed the data and provided comments on the manuscript. SS, AZ, and JW collected and processed the data. AB-S provided comments on the manuscript. CL and AP conceptualized the overarching project, contributed to the initial conceptualization of the manuscript, acquired funding, handled the project administration, and provided comments on the manuscript. All authors contributed to the article and approved the submitted version.

## Funding

This work was supported by the Department of Defense (W81XWH-16-1-0613), the National Heart, Lung and Blood Institute (R01 HL152740-1 and R01 HL128818-05), the National Heart, Lung and Blood Institute with National Institute of Aging (R01 HL128818-05 S1), the National Institute of Neurological Disorders and Stroke (K23 063371), the National Library of Medicine (5T15LM007059-27), the Pennsylvania Department of Health, the Mario Lemieux Foundation, and the Twenty Five Club Fund of Magee Women's Hospital.

## Conflict of Interest

The authors declare that the research was conducted in the absence of any commercial or financial relationships that could be construed as a potential conflict of interest.

## Publisher's Note

All claims expressed in this article are solely those of the authors and do not necessarily represent those of their affiliated organizations, or those of the publisher, the editors and the reviewers. Any product that may be evaluated in this article, or claim that may be made by its manufacturer, is not guaranteed or endorsed by the publisher.
